# A new non-human primate model of desiccating stress-induced dry eye disease

**DOI:** 10.1038/s41598-022-12009-7

**Published:** 2022-05-13

**Authors:** Li Gong, Yilin Guan, WonKyung Cho, Baowen Li, Lingzhen Pan, Zhenyan Yang, Mingling Wu, Zunyuan Yang, Sunil K. Chauhan, Wen Zeng

**Affiliations:** 1 PriMed Non-Human Primate Research Center of Sichuan PriMed Shines Bio-Tech Co., Ltd., Ya’an, 625000 Sichuan China; 2 Department of Ophthalmology, Harvard Medical School, Schepens Eye Research Institute of Massachusetts Eye and Ear, Boston, MA 02114 USA

**Keywords:** Drug discovery, Diseases

## Abstract

Dry eye disease (DED), a multifactorial ocular surface disease, is estimated to affect up to 34% of individuals over 50 years old. Although numerous animal models, including rodents and rabbits, have been developed to mimic the pathophysiologic mechanisms involved in dry eye, there is a lack of non-human primate (NHP) models, critical for translational drug studies. Here, we developed a novel desiccating stress-induced dry eye disease model using Rhesus macaque monkeys. The monkeys were housed in a controlled environment room for 21 to 36 days under humidity, temperature, and airflow regulation. Following desiccating stress, NHPs demonstrated clinical symptoms similar to those of humans, as shown by increased corneal fluorescein staining (CFS) and decreased tear-film breakup time (TFBUT). Moreover, corticosteroid treatment significantly reduced CFS scoring, restored TFBUT, and prevented upregulation of tear proinflammatory cytokines as observed in dry eye patients following steroid treatment. The close resemblance of clinical symptoms and treatment responses to those of human DED patients provides great translational value to the NHP model, which could serve as a clinically relevant animal model to study the efficacy of new potential treatments for DED.

## Introduction

Dry eye disease (DED), a highly prevalent multifactorial inflammatory disorder, results in symptoms of ocular discomfort, tear film instability, and visual disturbance^1–6^. Beyond hindering visual acuity, DED poses a great burden on the patient as it can result in continuous ocular discomfort, impacting daily activities and hampering one’s quality of life^7,8^. Depending on its etiology, DED is primarily classified into aqueous deficient dry eye disease (ADDE), resulting from reduced tear secretion, and evaporative dry eye disease (EDE)^9–11^. Both etiologies result in the disruption of the homeostasis of the tear film, which triggers a cascade of ocular inflammation and subsequent damage^2,3,10,11^. Tear film breakup time (TFBUT) and corneal fluorescent staining (CFS), measuring tear film instability and corneal epitheliopathy, respectively, are used to measure the extent of dry eye disease^2^.

Since being recognized as a disease in the 1990s, our understanding of the underlying pathophysiology of DED has greatly expanded. Ocular surface inflammation, especially perpetuated by T cells, damages the corneal barrier in DED^3,12^; thus, treatment has been focused on regulating the immune pathway to suppress corneal inflammation^1,11–13^. Despite the significant advancement in our understanding of DED and its extensive patient population, only few non-specific anti-inflammatory drugs with moderate efficacy and low tolerability are available for the treatment of DED^5,14^. The colossal discrepancy between the demand for DED-specific treatment and the development of such drugs is partly due to the limited informative animal models to study the translational drug efficacy^15,16^. A variety of experimental animal models have been developed to study the pathogenesis of DED; however, the species of choice have largely been limited to rodents and rabbits^16–18^. Although murine and rabbit models provide the benefit of wide availability and ease of manipulation, their anatomical, physiological, and immunological characteristics of the ocular surface differ from those of humans^15,19^. As the eyes of non-human primates (NHP) most closely resemble the human eye, Qin et al.^4^ have developed a severe dry eye disease model in rhesus monkeys by excising the lacrimal gland; however, this is an irreversible aqueous-deficient dry eye disease model, and currently, there is no NHP model of evaporative dry eye disease. Moreover, current animal models of EDE utilize both the dry eye chamber and atropine or scopolamine injection^14,16^, which concurrently suppresses immune cell infiltration^20^, hindering an accurate assessment of the pharmacodynamics in potential drug studies.

Given the greater prevalence of dry eye of EDE etiology^11^, and the need for an animal model that closely resembles humans, the purpose of our study was to develop a novel NHP model of EDE for the use of pharmacokinetic and pharmacodynamics studies. Here, we induced DED by exposing the NHPs only to the controlled environment room without the utilization of secondary pharmacology intervention. The monkeys developed DED, similar to those observed in human patients, as demonstrated by increased corneal fluorescein staining score, upregulation of inflammatory cytokines, and decreased TFBUT and tear volume.

## Results

### Controlled environment room

**Figure 1:**
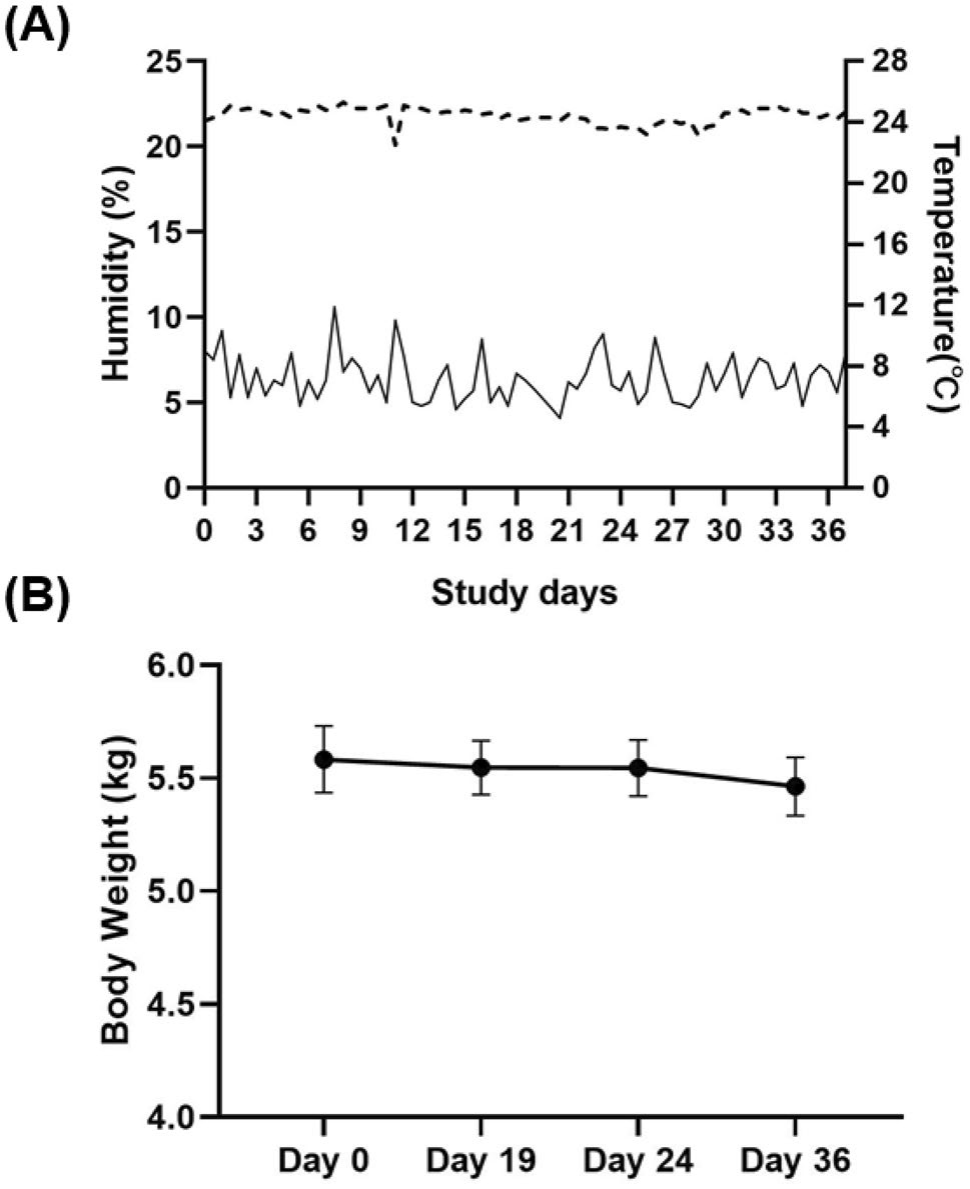
Monitoring of environmental conditions and body weight of the primates. (**A**) The relative humidity (%) and temperature of the controlled environment room throughout the study. (**B**) The body weight of the monkeys during the study.

During the induction of dry eye disease and treatment, the monkeys were housed in a controlled environment room with a relative humidity maintained at 6.4% ± 1.4% (Fig. 1A). The airflow was kept constant at 12 L/min. The temperature was maintained between 22.5 and 25.3 °C (Fig. 1A). The monkeys demonstrated normal behavior comparable to those housed in standard cages. No stress responses, including aggressive behavior, hair loss, and excessive eye rubbing, were observed. In addition, the body weight of the monkeys remained relatively stable throughout the course of the study (Fig. 1B).

### Dry eye induction induces corneal epitheliopathy

**Figure 2:**
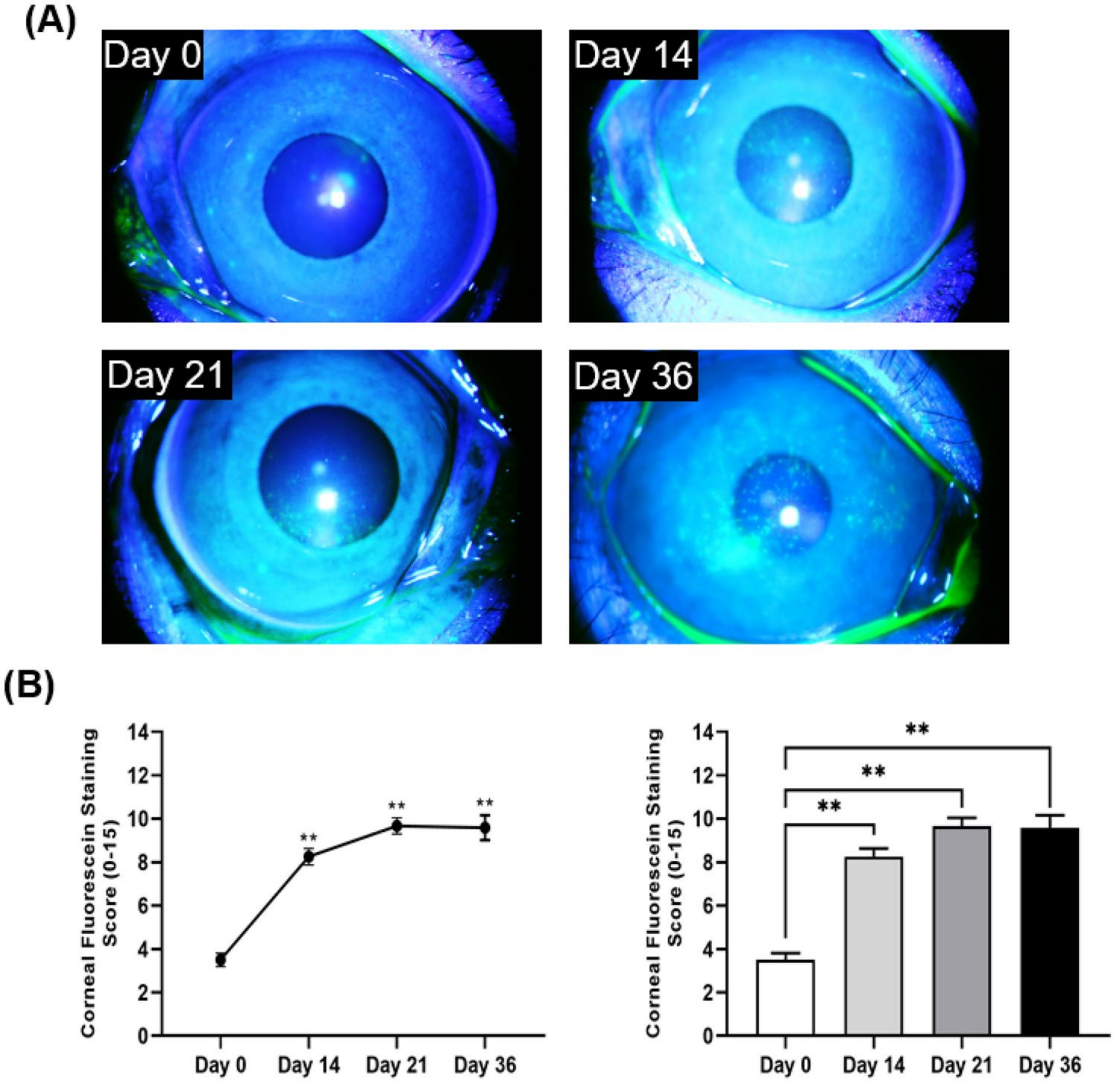
Corneal fluorescein staining of primate corneas. (**A**) Representative slit-lamp micrographs of fluorescein-stained corneas of monkeys housed in the controlled environment room on days 0, 14, 21, and 36. (**B**) Line graph (left) and bar chart (right) quantifying the corneal fluorescein score on indicated days post-dry eye induction. Representative data from two independent experiments are shown; each experiment consisted of 6 to 12 monkeys (n = 12–24 eyes/time point). Data are represented as mean ± SEM (error bar). T-test; *p < 0.05, **p < 0.01.

At baseline (day 0), corneas showed minimal or no punctate staining (3.5 ± 1.5) (Fig. 2A). A statistically significant increase in CFS score was observed on day 14 (8.3 ± 1.8, *p* < 0.001), which remained high through day 21 (9.7 ± 1.8, *p* < 0.001), and day 36 (9.6 ± 2.0, *p* < 0.001), compared to the baseline (Fig. 2B). Corneal staining was substantially higher in the central and nasal areas relative to other areas of the ocular surface.

### Exposure to controlled environment room induces tear film instability, inflammation and decreased tear secretion

**Figure 3:**
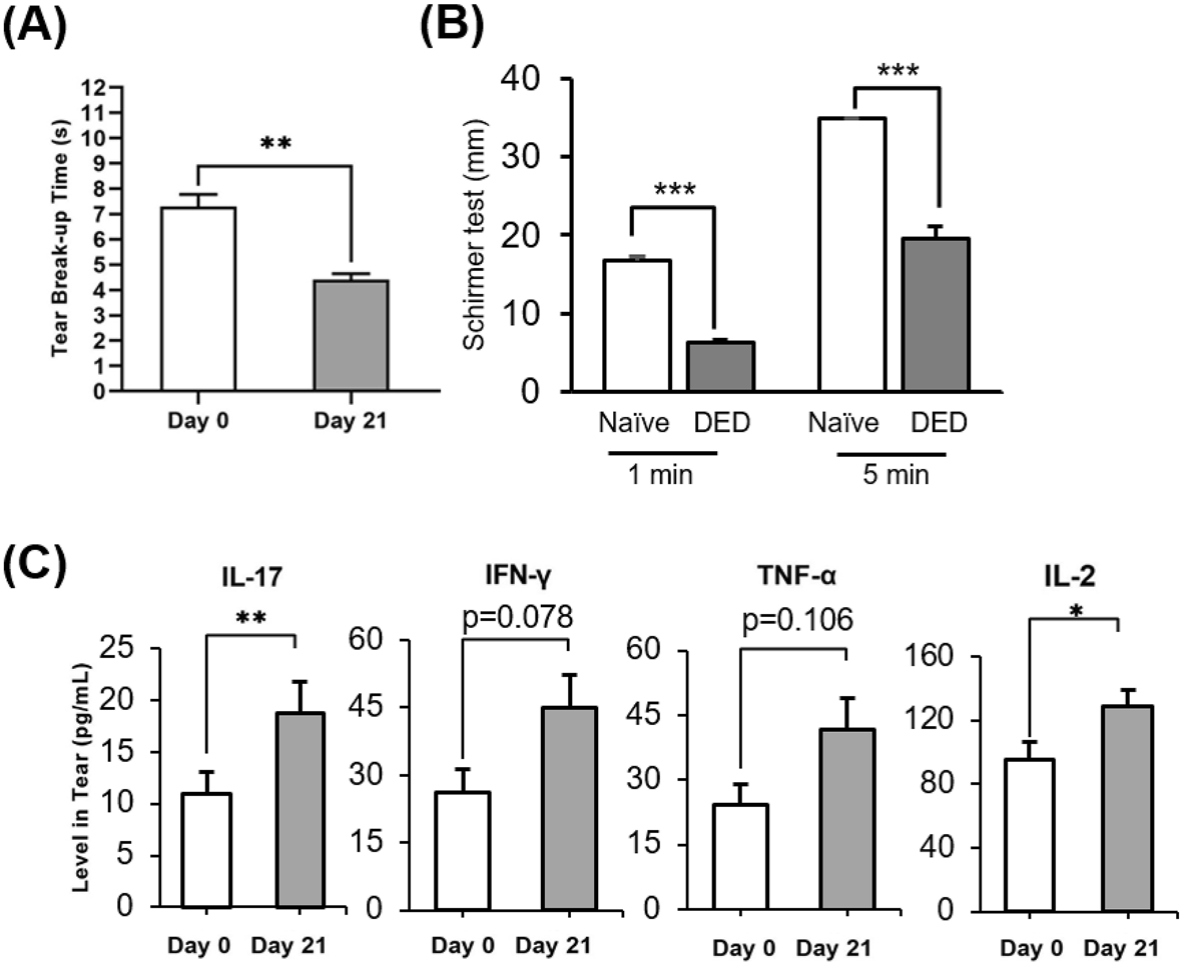
TFBUT, tear volume and cytokine levels in tears. (**A**) Bar chart showing TFBUT, measured by the time interval between complete blink and appearance of the first dry spot, on days 0 and 21 following controlled environment room exposure. (**B**) Bar chart quantifying tear volume in naïve and post-DED induction primates at 1 and 5 min of Schirmer test. (**C**) Tear wash was collected on days 0 and 21 of the controlled environment room housing (25 µL/wash) to measure the levels of proinflammatory cytokines, IL-17, IFN-γ, TNF-α, and IL-2. Representative data from two independent experiments are shown; each experiment consisted of 6 monkeys (n = 12 eyes). Data are represented as mean ± SEM (error bar). T-test; *p < 0.05, **p < 0.01, ***p < 0.001.

As clinical severity peaked and plateaued by day 21 of desiccating stress, subsequent studies housed the monkeys in the controlled environment room for 21 days to induce dry eye. Compared to baseline (Day 0; 7.3 ± 2.4 s), tear film break-up time decreased significantly on day 14 (5.3 ± 0.8 s, *p* = 0.007) and day 21 (4.4 ± 0.8 s, *p* < 0.001) following dry eye induction (Fig. 3A). Moreover, dry eye induction resulted in a significant downregulation in tear volume as measured by the Schirmer test at 1 min (6.2 ± 0.5 mm; *p* < 0.001) and 5 min (19.6 ± 1.6 mm, *p* < 0.001) compared to their naïve baseline control (16.7 ± 0.6 mm and > 35 mm, respectively) (Fig. 3B).

To measure ocular surface inflammation, tears from the ocular surface were collected on days 0 and 21 post-induction. Tears of monkeys housed in the controlled environment room showed a significant increase in levels of proinflammatory cytokines, IL-17 (*p* < 0.01) and IL-2 (*p* < 0.05), and a 1.5-fold increase in IFN-γ and TNF-α (Fig. 3C).

### Topical corticosteroid treatment suppresses dry eye-induced corneal epitheliopathy

To validate the therapeutic reversibility of the model, monkeys were randomized into two treatment groups after being housed in the controlled environment room for 21 days. Monkeys, continuously housed in the controlled environment room, were topically treated with Pred Forte 1% for 14 days and normal saline served as a control.

**Figure 4:**
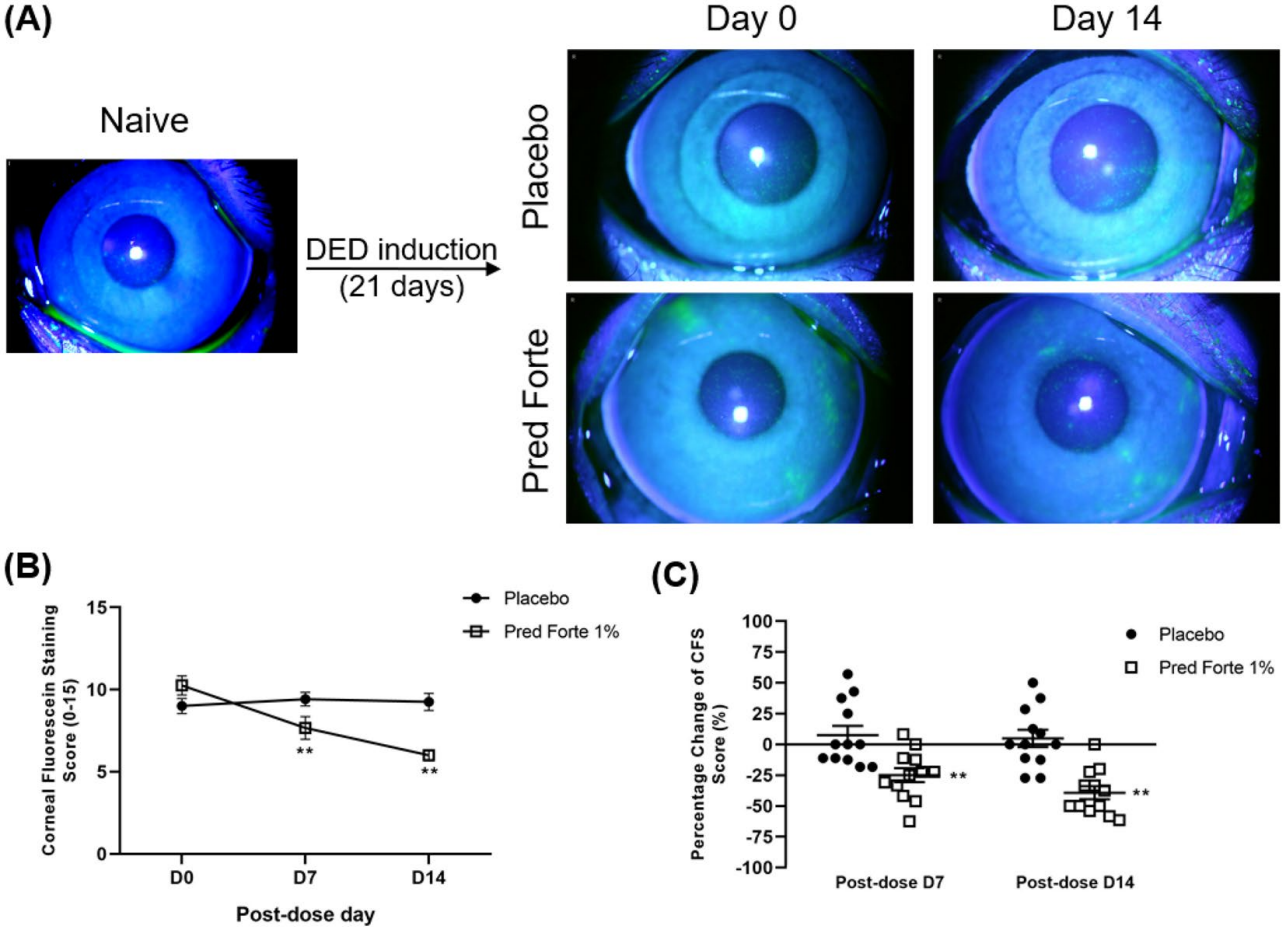
Corneal fluorescein staining following topical corticosteroid treatment. Rhesus monkeys were housed in a controlled environment room for 21 days to induce evaporative dry eye. From day 21 post-dry eye induction (Day 0 of treatment), both corneas were topically treated with two drops of Pred Forte 1% or normal saline (placebo) thrice daily for 14 days. (**A**) Representative corneal fluorescein staining images of Pred Forte 1% and placebo-treated monkeys on day of treatment initiation (Day 0), which serves as the baseline, and day 14 post-treatment induction. (**B**) Line graph showing CFS scores on days 0, 7, and 14 post-treatment induction. (**C**) Dot plot depicting percent change in CFS score from baseline (pre-treatment) on day 7 and 14 post-treatment induction. Each dot represents a single reading from a monkey. Representative data from two independent experiments are shown, each treatment group consisted of n = 6 eyes/group. Data are represented as mean ± SEM (error bar). T-test; **p < 0.01.

Comparable levels of CFS staining were observed before treatment (Day 0; Fig. 4A). Pred Forte 1% treated group showed a significant decrease in corneal staining compared with normal saline-treated group on day 7 (7.7 ± 2.4 vs. 9.4 ± 1.4, *p* = 0.041) and day 14 (6.0 ± 1.2 vs. 9.3 ± 1.8, *p* < 0.001) (Fig. 4B,C) of treatment.

### Topical corticosteroid treatment restores tear film stability and suppresses inflammation

**Figure 5:**
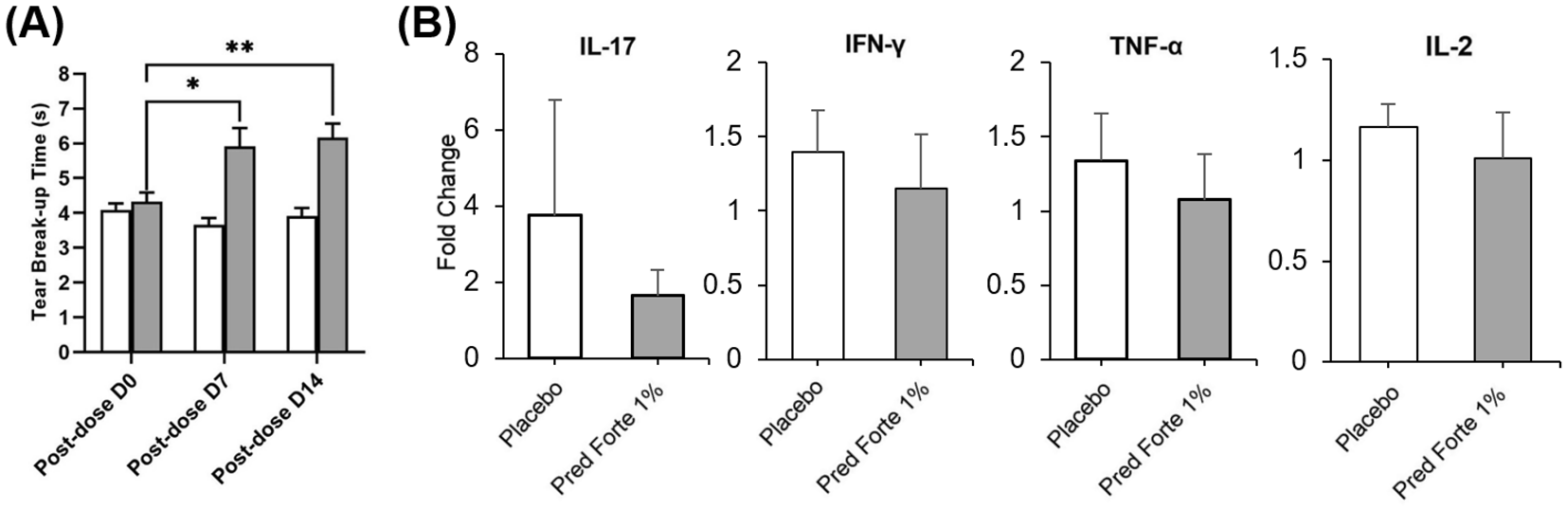
TFBUT and cytokine levels in tears following topical corticosteroid treatment. Rhesus monkeys were housed in a controlled environment room for 21 days to induce evaporative dry eye. From day 21 post-dry eye induction (Day 0 of treatment), both corneas were topically treated with two drops of Pred Forte 1% or normal saline (placebo) thrice daily for 14 days. (**A**) Bar chart showing TFBUT in Pred Forte 1% or placebo-treated corneas on days 0, 7 and 14 post-treatment initiation. (**B**) Tear wash (25 µL/wash) was collected on day 0 and 14 post-treatment initiation to measure the levels of proinflammatory cytokines, IL-17, TNF-α, IFN-γ and IL-2. Bar chart showing fold change in proinflammatory cytokines in tears of Pred Forte 1% or placebo-treated corneas on day 14 post-treatment. (Fold change from treatment day 0; 1 representing no change in cytokine levels). Representative data from two independent experiments are shown, each treatment group consisted of n = 6 eyes/group. Data are represented as mean ± SEM (error bar). t-test; *p < 0.05, **p < 0.01.

As DED is hallmarked by the disruption of tear film homeostasis and ocular surface inflammation, TFBUT and proinflammatory cytokines in tears were measured to assess the treatment efficacy of prednisone. TFBUT was restored to naïve levels of 7 s (Fig. 3A) following Pred Forte 1% on day 7, with no significant change in TFBUT following placebo treatment (6.1 ± 2.0 vs. 3.9 ± 0.6, *p* = 0.001) (Fig. 5A). Similar findings were observed on day 14 of treatment (6.1 ± 1.4 vs. 3.9 ± 0.8, *p* < 0.001) (Fig. 5A). Interestingly, no significant change in levels of tear inflammatory cytokines was observed following Pred Forte 1% treatment, compared to their pre-treatment baseline (post-dose day 0) (Fig. 5B). In contrast, cytokine levels continued to increase in the normal saline treatment group, suggesting prednisone treatment curbs the progression of inflammation at the ocular surface.

## Discussion

Dry eye disease is a significant public health issue^21–24^, affecting millions of patients in the US alone^9,25^. Here, we develop for the first time a non-human primate model of evaporative dry eye. Our findings demonstrate that by housing the monkeys in a controlled environment room of desiccating stress, the monkeys develop experimental DED, as evidenced by increased corneal epitheliopathy, reduced tear film breakup time, and ocular surface inflammation. This new model greatly advances the experimental tool of studying dry eye and its therapeutics as the non-human primates closely resemble the anatomical, physiological and immunological systems of humans^26,27^.

Due to its multifactorial etiologies and symptoms, numerous experimental dry eye animal models have been developed^4^. Although murine and rabbit models have been beneficial in studying the underlying pathophysiology of DED, distinctions in anatomical and physiological differences between mice and humans have limited translational research^15,27^. Thus, we developed a new experimental animal model utilizing non-human primates and a well-established dry eye model of desiccating stress. Since its first introduction in the early 2000s, the dry eye model of desiccating stress using a controlled environment chamber has been widely used to study evaporative dry eye disease^20,28^. Similarly, rhesus monkeys were housed in a controlled environment room with relative humidity below 15% and a constant airflow of 12 L/min. The monkeys did not exhibit any stress behaviors, and their body weight remained relatively constant throughout the entire study, demonstrating that the experimental conditions did not induce excessive stress. The animals developed clinical symptoms of DED by day 14 following desiccating stress induction, as shown by increased corneal epitheliopathy, decreased tear volume and disruption to tear film stability. As indicated by an increase in CFS score, significant corneal epitheliopathy was observed by day 14 remained high throughout the experiment until day 36. Moreover, to measure tear film instability, a hallmark of dry eye disease^9,29^, tear film breakup time was measured in the monkeys throughout the experimental conditions. Significant shortening in tear film breakup time was observed following desiccating stress exposure. Similar to the aqueous-deficient dry eye model in rhesus monkeys^4^, our EDE non-human primate model showed significant reduction in tear volume, as indicated by a two to threefold shorter Schirmer test readings following DED induction. In conjunction, a significant upregulation of proinflammatory cytokines was observed in tears. Our data clearly demonstrate that the controlled environment room effectively induces EDE in non-human primates.

The disparity between the growing DED patient population and the lack of effective targeted treatment is primarily due to the limited successful translation of preclinical studies to the clinic^15,30^. Given their high degree of resemblance in physiological responses and anatomy, non-human primate model is considered the gold standard of animal models for drug development and approval by the FDA^26^. In particular, the single main lacrimal gland and larger eye size^4^, similar to those of humans, make NHP an ideal model to study not only the therapeutic efficacy but the pharmacokinetics and effective drug delivery to the eye^15^. Despite its many benefits, only one NHP model of DED has been characterized due to the costs and need of a special facility to house non-human primates. Qin et al.^4^ have demonstrated that complete excision of the principal lacrimal gland results in severe dry eye disease. However, the lacrimal gland excision model is an effective model to study aqueous deficient dry eye disease and not EDE, which affects a larger population of dry eye patients^11^. Moreover, complete excision induces irreversible damage and is not an ideal model to study the progression and underlying mechanism of therapeutic improvement. In contrast, the new NHP model of desiccating stress-induced DED allows observation of pathophysiological reversal and progression of disease in response to treatment. Topical corticosteroid, a potent anti-inflammatory drug, reduces signs and symptoms of DED by suppressing inflammation^9^. To test the comparability of our NHP model to DED observed in humans, including therapeutic response, monkeys were treated with a known DED treatment of 1% prednisone. Following prednisone treatment, a significant improvement in CFS scoring was observed relative to the placebo treatment. Moreover, the treatment restored tear homeostasis and prevented the exacerbation of ocular inflammation, as exemplified by the normalization of TFBUT and the maintenance of tear inflammatory cytokine levels. Despite being exposed to continuous desiccating stress, we observed a trend of Pred Forte treatment controlling the upregulation of tear cytokine levels, suggesting that following more prolonged treatment and without continuous desiccating stress, our primates will have a similar therapeutic response to those seen in the clinic. Taken together, these data demonstrate that the NHP EDE model resembles not only the clinical symptoms and signs of EDE but also the therapeutic response, highlighting it as an effective model to study the therapeutic efficacy of new dry eye disease drugs in the preclinical setting.

One of the biggest limitations of using NHP experimental models is the ethical concerns^4^. Our model does not utilize any invasive manipulation of the monkeys, and the controlled environment room does not induce excessive stress, as exemplified by their stable weight and lack of abnormal behavior. In addition, the NHP model of the controlled environment room is superior to the murine model utilizing a controlled environment chamber not only in the anatomical similarity between the human and monkeys but in that the environment more closely resembles those of humans. When housed in their cages, mice are constantly exposed to the dust from the bedding and bodily fluid buildups in the cages. Such irritants can confound the pathogenesis and therapeutic efficacy but are absent in the controlled environment room NHP model.

Murine and rabbit models have enormously expanded our understanding of the pathophysiology of dry eye disease; however, due to cross-species differences, the existing models have limited efficacy in developing dry eye therapies^15^. We have successfully developed a new NHP model of evaporative dry eye disease, which shows comparable clinical symptoms and therapeutic response to patients with dry eye disease. As an effective preclinical validation model, the NHP model will provide an invaluable tool for novel drug development and vastly improve the efficacy of translational research.

## Materials and methods

### Animals

Twelve female Rhesus macaque monkeys (*M. mulatta*) between the age of 4 to 5 years were used in these experiments. Monkeys had free access to drinking water and were fed with monkey chow (12% calories from fat, 18% calories from protein, and 70% calories from carbohydrates; 200–300 g/day). In addition, daily allotment of fruits, vegetables, or additional supplements and various toys were also provided. All experimental protocols (AW2038) were reviewed and approved by the Institutional Animal Care and Use Committee (IACUC) of Sichuan Primed Shines Bio-tech Co., Ltd.. No animals were sacrificed for the purposes of this work. This study adhered to the tenets of the Declaration of Helsinki and complied with the National Institutes of Health Guide for the Care and Use of Laboratory Animals and the Association for Research of Vision and Ophthalmology guidelines.

### Induction of dry eye

The monkeys were housed in a controlled environment room with relative humidity (RH) below 15%, an airflow of 12 L/min, and at a temperature of 21 °C to 26 °C, for 36 consecutive days. Dry eye was clinically evaluated using corneal fluorescein staining on days 0, 14, 21, and 36 post-dry eye induction.

### Treatment regimen

Twenty-one days after the induction of dry eye, the monkeys in the controlled environment room were randomly divided into two treatment groups (n = 6 monkeys/12 eyes in each group): (1) a group receiving topical normal saline as placebo, (2) a group receiving topical Pred Forte 1% (Allergan, Inc., USA). Two drops of normal saline and Pred Forte 1% were topically applied to both eyes of unanesthetized monkeys three times a day for 14 days (total, 42 doses). Dry eye was clinically evaluated using fluorescein staining on treatment days 0, 7, and 14 (treatment day 0 equates to post-induction day 21).

### Corneal fluorescein staining (CFS)

Corneal fluorescein staining was performed on days 0 (baseline), 14, 21, and 36 after induction of dry eye and on days 0, 7, and 14 of the treatment regimen. Ten microliters of liquid 10% fluorescein (Alcon Laboratories Inc., USA) were applied to the inferior-lateral conjunctival sac of the monkey, and after 10 min, corneal fluorescein staining was examined under the cobalt blue light using a slit lamp biomicroscope (TOKA TSL-5, Wenzhou Raymond Photoelectricity Tech. Co., Ltd., China). Punctate staining was evaluated in a masked fashion using the National Eye Institute grading system, giving a score from 0 to 3 for each of the five areas of the cornea^17^.

### Tear film breakup time (TFBUT)

Tear film breakup time was measured following the guidelines outlined by the Report of the International Dry Eye Workshop (DEWS) 2017^11^. Ten microliters of preservative-free solution of 2% fluorescein was applied to the conjunctival sac with a micropipette. The monkeys were made to blink three times by an ophthalmologist to ensure adequate mixing of the dye. The time interval between the last complete blink and the appearance of the first corneal black spot, indicating disruption to the tear film, was measured using a stopwatch. The background illumination intensity was kept constant (cobalt blue light), and an integrated yellow filter was used to enhance the visibility of the tear film over the entire cornea. TFBUT was measured three times per eye, and the mean value of the measurements was calculated.

### Schirmer test

Schirmer I test following local anesthetic application was performed on days 0 (baseline), and 21 after induction of dry eye to measure the basic tear secretion in the monkeys as described previously^4^. In brief, a 35 mm Schirmer test strip (Eickemeyer, Tuttlingen, Germany) was inserted into the lower conjunctival fornix at the junction of the middle and lateral third of the lower eyelid margin. Eye lids were gently closed, and the extent of wetting was measured after 1 and 5 min, respectively.

### Tear collection

Monkeys were laid on a table in a supine position after being anesthetized with intramuscular injection of 10 mg/kg ketamine hydrochloride (Jiangsu Zhongmu Beikang Pharmaceutical Co., Ltd., China). Thirty microliters of phosphate-buffered saline were instilled into the inferior fornix, and monkeys were manually made to blink eight times. A total of 25 μL of tear wash was collected with a micropipette from the lateral canthus. To minimize ocular surface irritation, we collected the tear wash immediately after the application. The tear wash was placed into a 1.5 mL Eppendorf tube and stored at − 80 °C until further examination.

### Measurement of IL-17, IL-2, TNF-α, IFN-γ

LEGENDplex™ bead-based immunoassays (BioLegend, USA) were used to measure the levels of IL-17, IL-2, TNF-α, IFN-γ in tears according to the manufacturer’s instructions. Briefly, 25 μL of the solution composed of the tear, standard, mixed beads, and buffer solutions were added to each tube. Next, the tube was spun at 800 RPM and incubated for 2 h at room temperature. After three washes to remove the unbound proteins, 25 μL of corresponding detection antibodies were added to each tube. The tube was then spun at 800 RPM and incubated for another hour at room temperature. After three washes to remove the unbound detection antibodies, the samples were read on a flow cytometer (Beckman CytoFLEX, USA) and then analyzed using the LEGENDplex™ V8.0 software (BioLegend, USA).

### Statistical analysis

The groups were compared by paired or unpaired two-sample t-tests. All values are expressed as mean ± standard error of the mean (SEM). A p value < 0.05 was considered statistically significant. All statistical analysis was performed using Prism 9 software.
